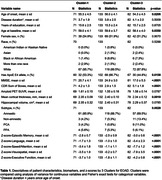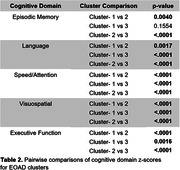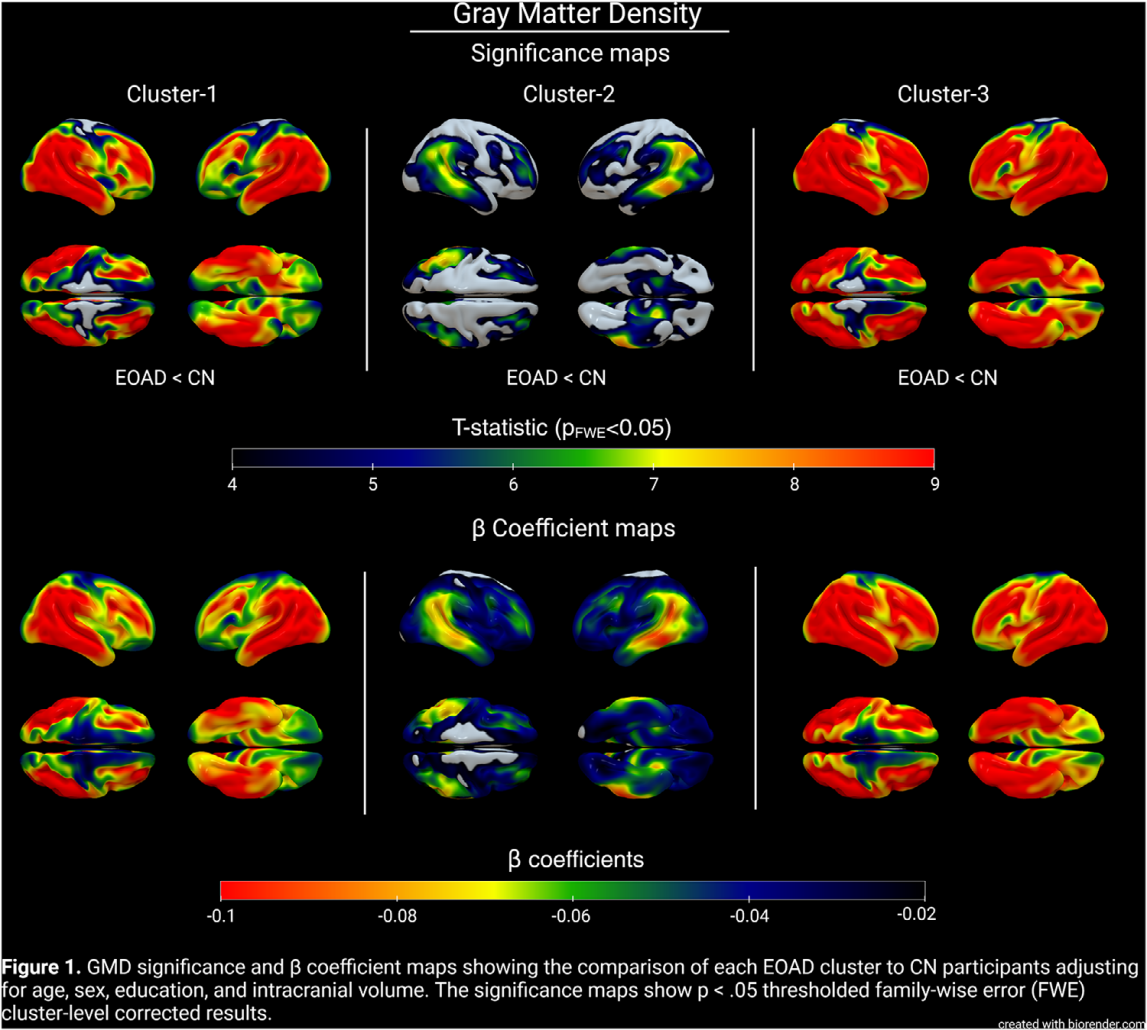# Cognitive clusters in sporadic early‐onset Alzheimer’s disease patients from the LEADS study

**DOI:** 10.1002/alz.095293

**Published:** 2025-01-09

**Authors:** Paige E. Logan, Kathleen A. Lane, Sujuan Gao, Ani Eloyan, Alexander Taurone, Maryanne Thangarajah, Alexandra Touroutoglou, Prashanthi Vemuri, Jeffrey L. Dage, Kelly N. Nudelman, Maria C. Carrillo, Bradford C. Dickerson, Gil D. Rabinovici, Liana G. Apostolova, Dustin B. Hammers, LEADS Consortium

**Affiliations:** ^1^ Indiana University School of Medicine, Indianapolis, IN USA; ^2^ Brown University, Providence, RI USA; ^3^ Massachusetts General Hospital and Harvard Medical School, Boston, MA USA; ^4^ Mayo Clinic, Rochester, MN USA; ^5^ Alzheimer’s Association, Chicago, IL USA; ^6^ Weill Institute for Neurosciences, University of California, San Francisco, San Francisco, CA USA

## Abstract

**Background:**

Early‐onset Alzheimer’s disease (EOAD) occurs before age 65 and has more diverse disease presentations than late‐onset AD. To improve our understanding of phenotypic heterogeneity among EOAD individuals, we analyzed cognitive scores using data‐driven statistical analysis.

**Method:**

Baseline cognitive data from 286 sporadic EOAD individuals from the Longitudinal EOAD study (LEADS) were transformed to z‐scores using data from 95 cognitively normal (CN) individuals. Cognitive composites were generated for domains of memory, language, speed/attention, visuospatial, and executive function. Residuals from linear regression models on Z‐scores adjusted for age, sex, and education were obtained. Cluster analysis using the Ward method on the cognitive domain residuals was performed and scree plot using the pseudo T‐squared determined the optimal number of clusters for the EOAD sample. We also compared gray matter density (GMD) of each EOAD cluster to CN participants using voxel‐wise multiple linear regressions.

**Results:**

Three clusters of cognitive performance were identified from the EOAD sample. Disease duration was not significantly different across clusters. Using a z‐score of ‐1.5 SD as the impairment threshold, all clusters were impaired across most domains (Table 1). Cluster‐3 was more impaired than cluster‐2 in all domains (Table 2; all p<.0001), and in all domains except episodic memory compared to cluster‐1 (all p<.01). Cluster‐1 (n = 71; 85.9% amnestic) was most impaired in executive function, visuospatial, and speed/attention. Cluster‐2 (n = 133; 88.7% amnestic) was most impaired in episodic memory. Cluster‐3 (n = 82; 69.5% amnestic) was most impaired in executive function, visuospatial, and speed/attention (Table 1). 3D‐comparisons showed all EOAD clusters had reduced GMD compared to CN. Cluster‐1 and cluster‐3 both showed widespread atrophy, with cluster‐3 being more severe. Cluster‐2 showed the most atrophy in the temporal and parietal lobes (Figure 1).

**Conclusion:**

We identified heterogeneity in cognitive patterns among sporadic EOAD individuals. Cluster‐3 appeared to reflect widespread impairment, and cluster‐2 represented an amnestic‐only presentation. Despite comparable disease duration, some EOAD patients progress faster, while some are more resilient. 3D‐comparisons showed neurodegenerative changes affecting brain regions responsible for respective impaired cognitive functions in each cluster (e.g., cluster‐2 is primarily amnestic‐impaired and has temporoparietal atrophy). Future work should explore amyloid‐PET and tau‐PET burden.